# Plant-derived Natural Products in Neurological and Psychiatric Disorders: Mechanisms of Action and Synergistic Roles with Probiotics

**DOI:** 10.1007/s12035-026-06065-7

**Published:** 2026-07-15

**Authors:** Mustafa M. Shokr, Abdelghafar M. Abu-Elsaoud, Seham M. Al Raish

**Affiliations:** 1 Department of Pharmacology and Toxicology, Faculty of Pharmacy, Sinai University – Arish Branch, Arish, 45511 Egypt; 2 Department of Biology, College of Science, Imam Mohammad Ibn Saud Islamic University (IMSIU), Riyadh, 11623 Kingdom of Saudi Arabia; 3 Department of Biology, College of Science, United Arab Emirates University, 15551, Al Ain, United Arab Emirates

**Keywords:** Therapeutics, Functional foods, Medicinal plants, Phytochemicals, SGLT2 inhibitors, Sustainable drug development

## Abstract

Plant-derived natural products have emerged as promising therapeutic agents for neurological and psychiatric disorders due to their diverse bioactive compounds and multi-target mechanisms of action. This review provides a mechanistic overview of medicinal plants and probiotics in modulating key pathological processes underlying disorders such as Alzheimer’s disease, epilepsy, autism spectrum disorder, and major depressive disorder. A systematic literature search was conducted across PubMed, Scopus, and Web of Science databases to identify relevant studies examining the neuroprotective, metabolic, and anti-inflammatory effects of plant-derived compounds, probiotics, and SGLT2 inhibitors. The findings highlight shared mechanisms, including attenuation of oxidative stress, suppression of neuroinflammation, modulation of mitochondrial function, and regulation of the gut–brain axis. Bioactive compounds such as polyphenols, flavonoids, alkaloids, and terpenoids demonstrate significant potential in improving neuronal survival and synaptic plasticity. Probiotics further contribute through microbiota-mediated regulation of neurotransmitters and immune responses. SGLT2 inhibitors are discussed as a comparative pharmacological model exhibiting overlapping mechanisms. Collectively, these integrative approaches provide a mechanistic framework for understanding how plant-derived compounds, probiotics, and metabolically active pharmacological models may influence shared pathways involved in neurological and psychiatric disorders. However, most proposed neuroprotective and synergistic effects require further validation through standardized formulations, well-designed clinical trials, and careful safety assessment before they can be translated into routine clinical practice.

## Introduction

Plant-derived natural products have emerged as promising therapeutic agents for neurological and psychiatric disorders due to their diverse bioactive compounds and multi-target mechanisms of action. Many of these compounds exert neuroprotective effects by modulating key pathological processes such as oxidative stress and chronic inflammation, which are central to disease progression [1]. By targeting multiple signaling pathways simultaneously, plant natural products offer a complementary approach to conventional therapies in managing complex neurological conditions. Neurological and psychiatric disorders are increasingly recognized as multifactorial conditions involving metabolic dysfunction, oxidative stress, and chronic inflammation [2]. Peripheral neuropathy is the most common presentation of diabetic neuropathy, which is a serious consequence of diabetes mellitus. The main cause of this illness is determined to be hyperglycemia [3, 4]. Marked axonal degeneration and segmental demyelination affecting peripheral sensory and motor nerves are hallmarks of the disease. Muscle atrophy and anomalies of the senses are the primary signs of the condition because damage to motor nerves can cause neuromuscular atrophy, which can result in reductions in muscle endurance, size, and metabolism [3, 5]. Numerous pathogenic processes, such as oxidative stress and neuroinflammation, can cause neurological impairment in people with diabetes. According to a recent study by Strom et al., diabetic neuropathy in diabetic individuals is substantially associated with lower levels of extracellular glutathione (GSH) and superoxide dismutase (SOD) [6]. Nevertheless, another study found that the erythrocytes of diabetic patients had much higher amounts of SOD and lower levels of GSH [7]. These somewhat contradictory experimental findings imply that diabetes patients have different levels of oxidative stress. These results provide credence to the idea that diabetic peripheral neuropathy is largely caused by oxidative stress. Additionally, high blood glucose levels may trigger the microvasculature's cyclooxygenase-2 pathway, which in turn encourages peripheral nerve inflammation and oxidative stress [8].

There is growing evidence that hyperglycemia contributes to BBB degradation in addition to its function in neurological impairment [9]. Inflammatory cytokines are upregulated as a result of hyperglycemia's promotion of reactive oxygen species (ROS), which in turn activate the signal transducer and activator of transcription (STAT) pathways, nuclear factor κ-light chain enhancer of activated B cells (NF-κB), and activation protein-1. Leukocyte extravasation, increased solute diffusion across the blood–brain barrier (BBB), and the entry of pathogens and toxins into the central nervous system are all made possible by the inflammatory response's disruption of BBB components like astrocytes and basement membranes, as well as the downregulation of expression of certain tight junction proteins like occludin and claudin-5 [10].

**Fig. 1:**
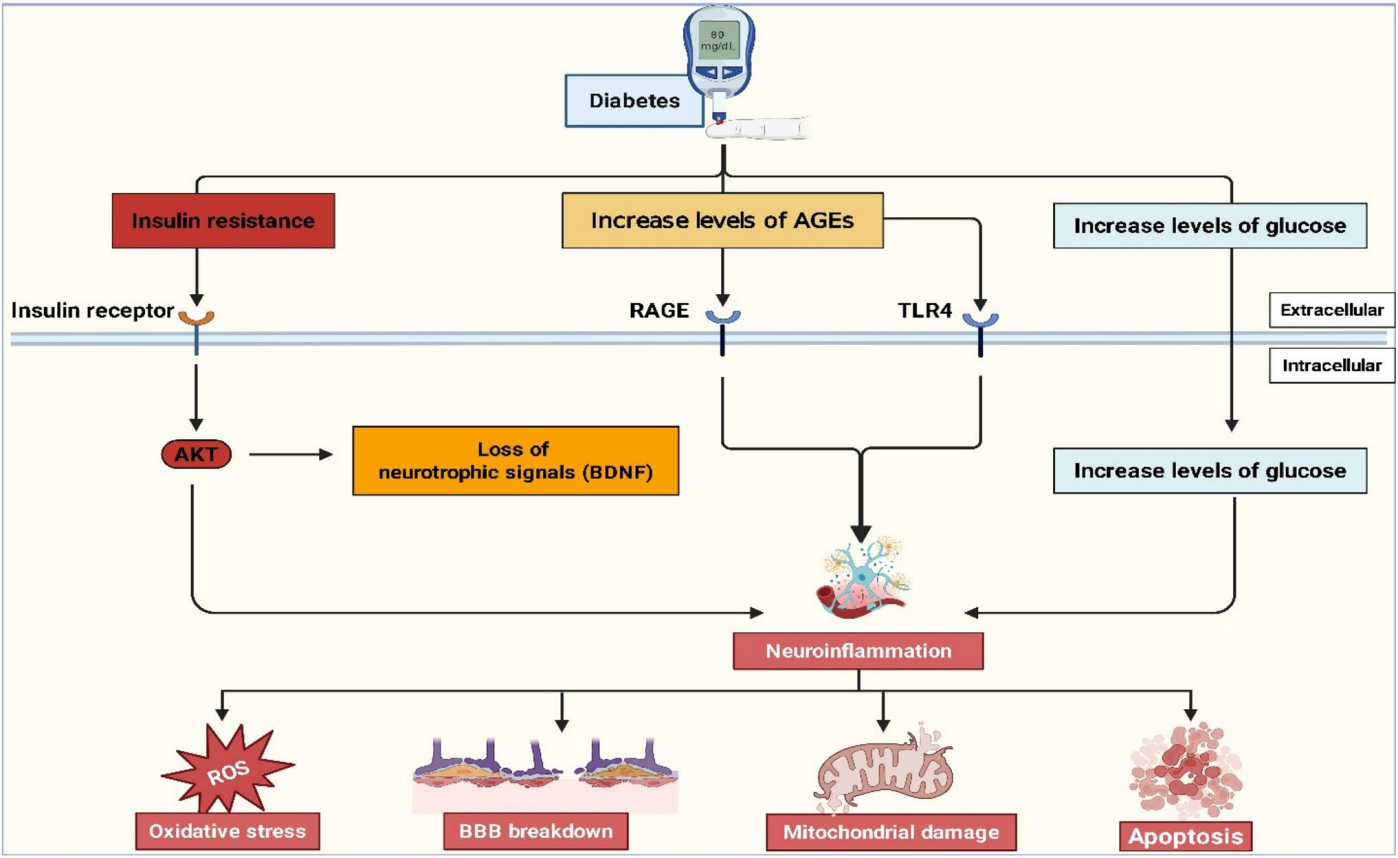
Pathophysiological mechanisms linking diabetes to neurological disorders. The central initiating factor is diabetes, leading to three primary downstream effects: (1) insulin resistance: impaired insulin signaling at the insulin receptor level leads to a loss of neurotrophic signals, particularly brain-derived neurotrophic factor (BDNF), mediated by the Akt pathway. This reduction in BDNF is critical for neuronal health and plasticity. (2) increased levels of advanced glycation end-products (AGEs): elevated AGEs interact with their receptors (RAGE) on cell surfaces, triggering intracellular signaling cascades. (3) Increased levels of glucose: High glucose levels, both extracellularly and intracellularly, directly contribute to cellular dysfunction. Intracellular glucose overload can activate toll-like receptor 4 (TLR4), further exacerbating inflammatory responses. These three primary diabetic complications converge to induce neuroinflammation. Neuroinflammation, in turn, drives a series of detrimental processes: (1) Oxidative stress, characterized by an increase in reactive oxygen species (ROS), which can damage cellular components. (2) Blood–brain barrier (BBB) breakdown: compromised integrity of the BBB allows harmful substances to enter the brain, further promoting inflammation and damage. (3) Mitochondrial damage: impaired mitochondrial function leads to energy deficits and increased cellular stress. (4) Apoptosis: Programmed cell death, resulting in neuronal loss and brain damage

Neuronal abnormalities are also associated with hyperglycemia. According to research, hyperglycemia raises dynamin-related protein 1 levels in neuronal mitochondria, impairing the structure and functionality of the mitochondria and ultimately causing synaptic dysfunction [11]. Furthermore, hyperglycemia causes an excess of ROS and free radicals, which speeds up neuronal death. Effector proteins are activated by the oxidative stress that follows, compromising the potential of the mitochondrial membrane and leading to increased permeability and swelling of the mitochondria. Consequently, the activation of the caspase-3 pathway facilitates the release of apoptogenic proteins, such as cytochrome c, from the mitochondria into the cytosol [12]. By increasing caspase activity, hyperglycemia further encourages apoptosis, which results in cellular processes such as DNA breakage, nuclear and structural protein disintegration, protein cross-linking, apoptotic body formation, and eventually phagocytic absorption [13] (Fig. 1).

Although several previous reviews have discussed medicinal plants or plant-derived phytochemicals in neurological disorders, the novelty of the present review lies in integrating plant-derived natural products, probiotics, and SGLT2 inhibitors within a shared mechanistic and translational framework. Rather than providing only a descriptive catalogue of medicinal plants, this review compares these interventions across convergent disease-relevant pathways, including oxidative stress, neuroinflammation, mitochondrial dysfunction, metabolic dysregulation, blood–brain barrier impairment, synaptic plasticity, and gut–brain axis signaling. This integrated perspective highlights how natural products and microbiota-based interventions may complement pharmacological models in targeting multifactorial neurological and psychiatric disorders, including Alzheimer’s disease, epilepsy, autism spectrum disorder, and major depressive disorder. Therefore, the main contribution of this review is its comparative focus on overlapping mechanisms and translational relevance, which distinguishes it from previous reviews that addressed medicinal plants, probiotics, or metabolic pharmacological agents separately.

SGLT2 inhibitors are included in this review not because they are plant-derived compounds, but because they provide a well-characterized comparative pharmacological model for metabolic, antioxidant, anti-inflammatory, vascular, and potential neuroprotective mechanisms. Their historical development from phlorizin, a naturally occurring plant-derived glucoside, also provides a relevant link between natural product chemistry and modern drug development. Therefore, SGLT2 inhibitors are discussed as a mechanistic reference point to contextualize whether medicinal plants and probiotics act on overlapping biological pathways, rather than being classified as botanical or plant-derived interventions.

## Methodology

### Study Design

This study was conducted as a narrative review with a systematic literature search to identify and synthesize current evidence on the roles of medicinal plants, probiotics, and SGLT2 inhibitors in neurological and psychiatric disorders. The review focuses on mechanistic insights and translational relevance across Alzheimer’s disease, epilepsy, autism spectrum disorder, and major depressive disorder.

### Literature Search Strategy

A comprehensive literature search was performed in PubMed, Scopus, and Web of Science databases for studies published up to May 2025. The search strategy combined Medical Subject Headings (MeSH) terms and keywords, including: “SGLT2 inhibitors,” “medicinal plants,” “phytochemicals,” “probiotics,” “gut–brain axis,” “Alzheimer’s disease,” “epilepsy,” “autism spectrum disorder,” and “major depressive disorder”. Boolean operators (AND, OR) were used to refine the search. Reference lists of relevant articles were also manually screened to identify additional eligible studies.

### Inclusion and Exclusion Criteria

Studies were included if they: were peer-reviewed original research articles (in vitro, in vivo, or clinical studies), were published in English, investigated the (neurological, psychiatric, or metabolic effects of medicinal plants, probiotics, or SGLT2 inhibitors), provided mechanistic insights or clinically relevant outcomes.

Studies were excluded if they: were conference abstracts, editorials, or case reports without original data, were not related to neurological or psychiatric disorders, lacked mechanistic or outcome-based findings, were duplicate records.

### Study Selection and Data Extraction

Two independent reviewers independently screened the titles and abstracts of all records retrieved from PubMed, Scopus, and Web of Science after duplicate removal. During the first screening stage, studies were excluded if they were clearly unrelated to neurological or psychiatric disorders, did not investigate medicinal plants, plant-derived bioactive compounds, probiotics, or SGLT2 inhibitors, or did not report mechanistic or clinically relevant outcomes. Articles that appeared potentially eligible were retrieved in full text and assessed against the predefined inclusion and exclusion criteria. Full-text studies were retained when they provided original experimental or clinical data relevant to neuroprotection, metabolic regulation, oxidative stress, neuroinflammation, mitochondrial function, blood–brain barrier integrity, synaptic plasticity, or gut–brain axis modulation. Studies were excluded at the full-text stage if they lacked sufficient mechanistic detail, did not include relevant neurological or psychiatric outcomes, were not original peer-reviewed articles, or duplicated data from another included report. Any disagreement between the two reviewers was resolved by discussion until consensus was reached.

Data extracted from the included studies comprised the study design, experimental model or human population, intervention type, investigated plant-derived compound, probiotic strain, or SGLT2 inhibitor, neurological or psychiatric outcome, metabolic outcome where applicable, proposed molecular mechanism, and level of evidence, including whether the findings were derived from in vitro, animal, or human clinical studies.

### Data Synthesis

Findings were synthesized narratively and organized into three thematic domains: (1) SGLT2 inhibitors, (2) medicinal plants and phytochemicals, and (3) probiotics and the gut–brain axis. During synthesis, each finding was classified according to the source of evidence as preclinical evidence, human clinical evidence, or mixed evidence. Preclinical evidence included in vitro experiments and animal disease models, whereas human clinical evidence included randomized controlled trials, pilot clinical trials, and observational studies involving human participants. When a mechanism was mainly supported by animal or in vitro studies but accompanied by limited human outcome data, it was classified as mixed evidence. This classification was incorporated into the revised tables and narrative interpretation to distinguish mechanistic findings from clinically validated effects and to avoid overgeneralizing preclinical data to human neurological practice.

In addition, the strength of evidence for each medicinal plant and probiotic intervention was summarized descriptively. Because this review used a narrative synthesis supported by a systematic literature search rather than a formal meta-analysis, evidence strength was classified according to the type and consistency of available studies. Evidence was considered strong when supported by multiple consistent human randomized controlled trials or meta-analyses; moderate when supported by at least one human clinical study together with consistent preclinical or mechanistic evidence; limited when based on small clinical studies, pilot trials, observational evidence, or inconsistent clinical findings; and preclinical or indirect when evidence was derived mainly from animal or in vitro studies or from non-neurological metabolic outcomes. This classification was used to distinguish clinically supported effects from mechanistic or preliminary findings.

### Study Selection Overview

**Fig. 2:**
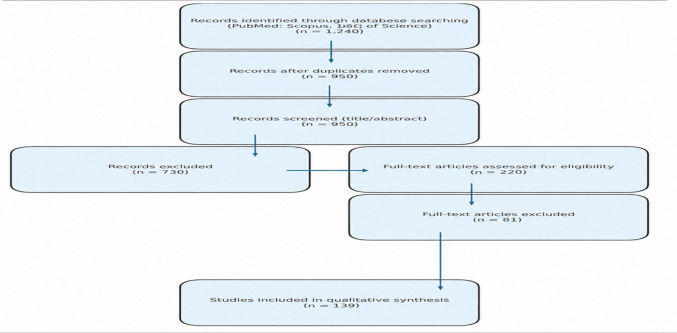
PRISMA 2020 flow diagram showing identification, duplicate removal, title and abstract screening, full-text eligibility assessment, exclusion reasons, and final inclusion of 80 studies in the qualitative synthesis

The literature search initially identified records from PubMed, Scopus, and Web of Science. After duplicate removal, 950 records were screened by title and abstract. At this stage, 730 records were excluded because they were not directly related to neurological or psychiatric disorders, did not examine medicinal plants, plant-derived compounds, probiotics, or SGLT2 inhibitors, were not original research articles, or did not report mechanistic or outcome-based findings relevant to the scope of the review. The remaining 220 articles were retrieved and assessed in full text. Of these, 81 articles were excluded because they lacked sufficient mechanistic detail, did not provide relevant neurological or psychiatric outcomes, focused only on general metabolic or pharmacological effects without neurobiological relevance, were review articles, editorials, conference abstracts, case reports, or contained overlapping data already represented by a more complete study. Finally, 139 studies met the eligibility criteria and were included in the qualitative synthesis. These 139 studies were selected because they provided relevant evidence on the neuroprotective, metabolic, anti-inflammatory, antioxidant, mitochondrial, synaptic, vascular, or gut–brain axis-related effects of medicinal plants, plant-derived compounds, probiotics, or SGLT2 inhibitors. The study selection process is illustrated in the PRISMA 2020 flow diagram shown in Fig. 2.

## SGLT2 Inhibitors: Chemistry, Absorption, and Pharmacological Characteristics

SGLT2 inhibitors have transformed type 2 diabetes mellitus (T2DM) management. Derived from phlorizin, they are aryl-C-glucosides that inhibit renal glucose reabsorption and induce glucosuria [14]. Modern agents (empagliflozin, dapagliflozin, canagliflozin, bexagliflozin) resist intestinal hydrolysis, improving bioavailability and SGLT2 selectivity (> 250-fold vs. SGLT1; up to 2,700-fold for empagliflozin) and reducing gastrointestinal effects [14, 15].

They are rapidly absorbed (Cmax 1–3 h), show dose-proportional pharmacokinetics unaffected by food, and have moderate protein binding with wide tissue distribution [14, 16]. Their C-aryl glycoside structure prevents enzymatic degradation, with metabolism via hepatic glucuronidation (UGT1A9, UGT2B7) and elimination in urine and feces [17].

SGLT2 inhibitors act in the proximal tubule (S1/S2), blocking ~ 90% of glucose reabsorption, lowering the renal threshold (~ 180 to 70–90 mg/dL), and increasing urinary glucose excretion (70–100 g/day) independently of insulin [15, 18]. This leads to reduced plasma glucose, weight loss, blood pressure reduction, and lipid profile modulation [19, 20].

They also promote ketogenesis (↑β-hydroxybutyrate) and exert extra-renal effects, including reduced oxidative stress and inflammation (NF-κB, TNF-α, IL-6), with potential actions in the heart, brain, and endothelium [21, 22]. Despite low brain expression, imaging suggests neuromodulatory roles [14, 23]. Clinically, they reduce heart failure hospitalization and slow diabetic kidney disease progression, with generally good safety; common effects include volume depletion and genital infections, while serious events are rare [15, 24, 25].

In conclusion, SGLT2 inhibitors combine distinct structure, predictable pharmacokinetics, and multifaceted mechanisms, supporting their role as foundational therapies for T2DM and emerging indications such as NAFLD and HFpEF [26, 27] (Table 1).

**Table 1:** Pharmacological and neuroprotective mechanisms of SGLT2 inhibitors

Mechanism	Key effects	Neurological relevance	Main evidence source	Ref
Glucose regulation	↓ renal glucose reabsorption, ↑ glucosuria	Improves systemic metabolic dysfunction; neurological relevance is mainly indirect	Human clinical evidence for metabolic outcomes; indirect neurological relevance	[15, 18]
Ketogenesis	↑ β-hydroxybutyrate	May enhance neuronal energy efficiency and reduce excitability	Human metabolic evidence with mainly preclinical neurological interpretation	[21, 26, 28]
Anti-inflammatory effects	↓ TNF-α, IL-6, NF-κB	May reduce neuroinflammatory signaling in AD, epilepsy, ASD, and MDD	Mainly preclinical and mechanistic evidence for neurological outcomes	[29–32]
Antioxidant effects	↑ SOD, ↑ GSH, ↓ ROS	Protects neurons from oxidative injury	Mainly preclinical and mechanistic evidence	[26, 33, 34]
Mitochondrial protection	↑ ATP production, improved mitochondrial function	May prevent neuronal apoptosis and support cellular resilience	Mainly preclinical evidence	[35]
BBB and vascular protection	Improved endothelial function, ↓ permeability	May help maintain CNS homeostasis	Mainly preclinical and mechanistic evidence; clinical vascular benefits are indirect	[36, 37]

## Shared Mechanistic Pathways in Neurological and Psychiatric Disorders: Insights from SGLT2 Inhibitors

Neurological and psychiatric disorders, including Alzheimer’s disease (AD), epilepsy, autism spectrum disorder (ASD), and major depressive disorder (MDD), share overlapping pathophysiological mechanisms such as oxidative stress, neuroinflammation, mitochondrial dysfunction, metabolic dysregulation, and blood–brain barrier (BBB) impairment. These interconnected pathways contribute to neuronal dysfunction, synaptic loss, and progressive neurodegeneration. Increasing evidence suggests that sodium-glucose cotransporter-2 (SGLT2) inhibitors exert pleiotropic effects that target these shared mechanisms, providing a valuable pharmacological framework for understanding integrative therapeutic strategies.

### Neuroinflammation

Chronic neuroinflammation is a central feature across multiple neurological disorders, characterized by activation of microglia and astrocytes and increased production of pro-inflammatory cytokines such as TNF-α, IL-6, and IL-1β. In Alzheimer’s disease, neuroinflammation exacerbates amyloid-beta (Aβ) toxicity and tau pathology, promoting neuronal damage and cognitive decline [38, 39]. Similarly, in epilepsy, inflammatory mediators contribute to neuronal hyperexcitability and seizure propagation through activation of the NLRP3 inflammasome and disruption of neuronal homeostasis [40, 41]. In ASD and MDD, elevated inflammatory cytokines and chronic immune activation are associated with impaired synaptic function, altered neurotransmitter metabolism, and behavioural abnormalities [31, 42].

SGLT2 inhibitors have demonstrated potent anti-inflammatory effects by suppressing microglial activation and inhibiting key signaling pathways such as nuclear factor κB (NF-κB), resulting in reduced production of pro-inflammatory cytokines [29–31, 43]. These effects contribute to attenuation of neuroinflammatory cascades, preservation of neuronal integrity, and improvement of disease-related outcomes across multiple neurological conditions.

### Oxidative Stress and Mitochondrial Dysfunction

Oxidative stress, driven by excessive production of reactive oxygen species (ROS), plays a critical role in neuronal injury and neurodegeneration. In AD, ROS accumulation contributes to Aβ aggregation, mitochondrial dysfunction, and neuronal apoptosis [44, 45]. In epilepsy, seizure activity induces oxidative damage to lipids, proteins, and DNA, further impairing neuronal function and increasing seizure susceptibility [46, 47]. Similarly, ASD and MDD are associated with increased oxidative stress and reduced antioxidant capacity, leading to mitochondrial dysfunction and impaired neuronal resilience [48, 49].

SGLT2 inhibitors mitigate oxidative stress by reducing ROS production and enhancing endogenous antioxidant defences, including superoxide dismutase (SOD) and glutathione (GSH) [26, 33, 34]. Additionally, they improve mitochondrial function by restoring membrane potential, increasing ATP production, and reducing mitochondrial swelling and dysfunction [35]. These effects collectively protect neurons from oxidative damage and promote cellular survival across neurological disorders.

### Metabolic Dysregulation and Ketogenesis

Metabolic dysfunction, particularly impaired glucose utilization and insulin resistance, is increasingly recognized as a contributing factor in neurological disorders. In AD, cerebral glucose hypometabolism is a hallmark feature, leading to impaired neuronal energy supply and cognitive decline [50]. In epilepsy, altered energy metabolism contributes to neuronal hyperexcitability, while in ASD and MDD, mitochondrial dysfunction and metabolic imbalance further exacerbate disease pathology [51, 52].

SGLT2 inhibitors improve metabolic homeostasis by reducing renal glucose reabsorption and promoting glucosuria, leading to decreased plasma glucose levels and improved insulin sensitivity [15, 18]. Importantly, they induce a metabolic shift toward ketogenesis, increasing levels of β-hydroxybutyrate, an alternative energy substrate that enhances neuronal efficiency and reduces excitability [21, 26, 28]. This mechanism parallels the therapeutic effects of ketogenic diets in epilepsy and provides a neuroprotective advantage in conditions characterized by impaired glucose metabolism.

### Synaptic Plasticity and Neurotransmission

Disruption of synaptic plasticity and neurotransmitter balance is a key feature of neurological and psychiatric disorders. In AD, synaptic loss and impaired cholinergic signaling contribute to cognitive decline [53, 54]. In epilepsy, imbalances between excitatory (glutamate) and inhibitory (GABA) neurotransmission lead to neuronal hyperexcitability and seizure activity [55]. ASD is characterized by altered excitatory/inhibitory (E/I) balance and synaptic dysfunction, while MDD is associated with reduced levels of brain-derived neurotrophic factor (BDNF) and impaired neuroplasticity [56, 57].

SGLT2 inhibitors enhance synaptic function by increasing BDNF expression, promoting neurogenesis, and improving synaptic plasticity [26, 58]. Additionally, they modulate neuronal excitability by regulating sodium influx and stabilizing neuronal membranes, thereby reducing hyperexcitability and abnormal neuronal firing [59]. These effects contribute to improved cognitive function, mood regulation, and seizure control.

### Blood–brain Barrier Integrity and Neurovascular Function

The integrity of the blood–brain barrier (BBB) is essential for maintaining central nervous system homeostasis. Disruption of the BBB allows infiltration of inflammatory mediators, toxins, and pathogens, contributing to neurodegeneration. In AD, BBB breakdown is associated with increased Aβ deposition and neuroinflammation [9, 10]. In epilepsy, BBB dysfunction facilitates seizure propagation and neuronal injury [37]. Similarly, BBB impairment is implicated in ASD and MDD, where it contributes to neuroinflammation and altered neuronal signaling [60, 61].

SGLT2 inhibitors improve BBB integrity and neurovascular function by reducing inflammation, enhancing endothelial function, and promoting cerebral blood flow [36, 37]. These vascular protective effects help maintain neuronal homeostasis, reduce neuroinflammatory infiltration, and support overall brain health.

### Translational Implications

The shared mechanisms underlying neurological and psychiatric disorders highlight the importance of multi-target therapeutic strategies. Increasing evidence indicates that oxidative stress, neuroinflammation, mitochondrial dysfunction, and metabolic dysregulation are common pathological features across conditions such as Alzheimer’s disease, epilepsy, autism spectrum disorder, and major depressive disorder [26, 29, 33, 36]. Targeting these interconnected pathways is therefore essential for developing effective and comprehensive therapeutic approaches.

SGLT2 inhibitors, through their combined anti-inflammatory, antioxidant, metabolic, and neuroprotective effects, provide a valuable pharmacological model that parallels the mechanisms of plant-derived natural products and probiotics. These agents have been shown to reduce oxidative stress, suppress pro-inflammatory signaling pathways such as NF-κB, enhance mitochondrial function, and improve neuronal survival [26, 29, 34]. Similarly, plant-derived bioactive compounds, including polyphenols, flavonoids, and alkaloids, exert comparable effects by modulating oxidative stress and inflammatory pathways, as well as regulating key signaling mechanisms involved in apoptosis and neuroprotection [62–70].

In addition, probiotics contribute to this integrative framework through modulation of the gut–brain axis, influencing neurotransmitter production, immune responses, and metabolic homeostasis [71–75]. These complementary mechanisms further support the concept of a multi-target approach, in which metabolic, inflammatory, and neurochemical pathways are addressed simultaneously.

It is important to distinguish the level of evidence supporting these findings. For SGLT2 inhibitors, the cardiometabolic, renal, and vascular benefits are supported by strong human clinical evidence; however, their direct neurological and neuroprotective effects remain largely supported by preclinical, mechanistic, or indirect clinical evidence. Similarly, several plant-derived compounds and probiotics show robust antioxidant, anti-inflammatory, mitochondrial, and neurotransmitter-modulating effects in animal models, but only selected interventions have been evaluated in human neurological or psychiatric populations. Therefore, the translational relevance of these interventions should be interpreted according to whether the evidence is derived from animal studies, mechanistic studies, or human clinical trials.

This mechanistic overlap supports the biological plausibility of integrative therapeutic strategies involving plant-derived natural products, probiotics, and pharmacological agents such as SGLT2 inhibitors. However, the available evidence should be interpreted cautiously. At present, there is insufficient direct clinical evidence to confirm true synergistic effects between medicinal plants, probiotics, and SGLT2 inhibitors in neurological or psychiatric disorders. Most support for synergy is based on shared mechanisms, preclinical findings, indirect metabolic evidence, or separate clinical studies of individual interventions rather than randomized trials testing combined regimens. Therefore, the proposed synergistic role should be considered a hypothesis-generating framework and a future research direction, not an established clinical recommendation. Well-designed randomized controlled trials are needed to evaluate whether combined interventions provide additive or synergistic benefits, determine optimal dosing and sequencing, and assess long-term safety, herb–drug interactions, and microbiota–drug interactions.

## Medicinal Plants and Probiotics with Dual Effects on Glucose–sodium Homeostasis and Neurological Disorders

An increasing number of natural products exhibit pleiotropic effects that may modulate metabolic pathways, oxidative stress, inflammation, and neurological signaling [76, 77]. These mechanisms partially overlap with pathways targeted by conventional pharmacological agents, including SGLT2 inhibitors, antiepileptic drugs, and antidepressants [78, 79]. This overlap is relevant to patients with comorbid metabolic and neurological or psychiatric conditions, such as type 2 diabetes mellitus, hypertension, epilepsy, Alzheimer’s disease, and major depressive disorder [80, 81]. However, the clinical relevance of these dual effects remains variable across interventions and is often supported by preclinical, indirect, or limited clinical evidence. Therefore, plant-derived natural products should currently be considered potential adjunctive or investigational approaches rather than established replacements for conventional evidence-based therapies.

Compared with conventional pharmacological approaches such as SGLT2 inhibitors, plant-derived natural products may offer broader multi-target mechanistic profiles, but their clinical translation is limited by variability in formulation, dose, bioavailability, safety monitoring, and strength of human evidence.

### Medicinal Plants with Dual Activity

#### *Salvia officinalis* (Sage)

Clinical trials and preclinical studies have demonstrated that *Salvia officinalis* improves glycemic control by enhancing insulin sensitivity and inhibiting α-glucosidase activity. Its essential oils also modulate GABAergic transmission and reduce oxidative stress, thereby conferring anticonvulsant and cognitive benefits [82–84]. These findings suggest that *Salvia officinalis* has the potential to be a valuable therapeutic option for individuals with neurological disorders such as epilepsy and cognitive impairments. Further research is needed to fully understand the mechanisms by which *Salvia officinalis* exerts its effects on neurological pathways and to optimize its use in clinical settings. Overall, the dual activity of medicinal plants like *Salvia officinalis* highlights the importance of exploring natural remedies in the treatment of neurological disorders. The neuroprotective properties of *Salvia officinalis* are linked to its antioxidant and anti-inflammatory activities, including modulation of cholinergic function and oxidative stress pathways. However, clinical evidence remains limited, and further studies are needed to establish its efficacy in neurological disorders [82–84].

#### *Nigella sativa *(Black Seed)

The principal bioactive constituent of *Nigella sativa*, thymoquinone, has been widely investigated for its antidiabetic and neuroprotective properties. Mechanistically, thymoquinone enhances glucose metabolism through the activation of AMPK, attenuation of oxidative stress, and reduction of systemic inflammation [62]. Clinical studies in patients with T2DM reported significant reductions in fasting blood glucose and glycated hemoglobin (HbA1c) following supplementation with *Nigella sativa* seeds or oil [63]. Preclinical evidence further supports its role as an anticonvulsant, with thymoquinone demonstrating protective effects against pentylenetetrazol (PTZ)-induced seizures and enhancing endogenous antioxidant capacity [64, 65]. A pilot clinical trial in children with intractable epilepsy indicated that adjunctive use of thymoquinone improved seizure control [66]. Collectively, these data suggest that *Nigella sativa* may represent a promising integrative agent for patients with comorbid diabetes and epilepsy, although larger-scale randomized controlled trials are warranted. Its neuroprotective effects are largely mediated through thymoquinone, which inhibits NF-κB signaling and enhances antioxidant defenses, thereby reducing oxidative stress and inflammation. However, its clinical application remains limited by insufficient large-scale human studies and variability in formulation [62–66].

#### *Moringa oleifera *(Moringa)

*Moringa oleifera* leaves contain diverse phytochemicals, including flavonoids and glucosinolates, which contribute to their potent antidiabetic activity. Preclinical investigations consistently show reductions in hyperglycemia, enhanced insulin sensitivity, and attenuation of oxidative stress [85]. Evidence from animal models of epilepsy has revealed anticonvulsant and anxiolytic properties, likely mediated by increased GABA activity and reduced ROS [86, 87]. Clinical data, though limited, suggest potential benefits of *Moringa* supplementation on glycemic indices and blood pressure in T2DM patients, with favorable safety profiles [88]. These findings highlight the therapeutic promise of *Moringa oleifera* as a multifunctional botanical, though rigorous long-term human studies remain necessary to validate efficacy and establish optimal dosing. The beneficial effects of *Moringa oleifera* are associated with its rich phytochemical content, which modulates oxidative stress and inflammatory pathways while supporting neuronal resilience. Nevertheless, further clinical validation and standardized dosing strategies are required to confirm its therapeutic potential [85, 86, 88].

#### *Curcuma longa* (Turmeric)

Curcumin, the primary polyphenol of *Curcuma longa*, exerts pleiotropic metabolic and neuroprotective actions. By activating AMPK and modulating inflammatory pathways such as NF-κB, curcumin improves insulin sensitivity and reduces circulating pro-inflammatory cytokines [67]. Clinical studies further demonstrate curcumin’s efficacy in reducing depressive symptoms in patients with major depressive disorder, supporting its role as an adjunct to conventional antidepressants [68]. Moreover, bioavailable curcumin formulations have shown promise in reducing amyloid-β aggregation and tau hyperphosphorylation, thereby ameliorating cognitive decline in older adults [69]. Despite heterogeneity in trial outcomes, systematic reviews confirm curcumin’s overall potential as a dual-action agent for metabolic and neurological disorders, though variability in formulation and bioavailability remains a limiting factor [89]. Curcumin exerts its effects through inhibition of NF-κB and activation of antioxidant-response pathways, leading to reduced inflammation and oxidative damage in neuronal tissues. Despite strong preclinical evidence, its clinical use remains limited by poor bioavailability and rapid metabolism [67–70].

#### *Hibiscus sabdariffa* (Roselle)

The calyces of *Hibiscus sabdariffa* are rich in anthocyanins and hibiscus acid, compounds known to exert antihypertensive, natriuretic, and antidiabetic effects. Clinical evidence supports significant reductions in both systolic and diastolic blood pressure, in part via angiotensin-converting enzyme inhibition [90, 91]. Randomized trials have also reported improvements in fasting glucose, lipid profiles, and endothelial function, indicating broader cardiometabolic benefits [92, 93]. Given its capacity to enhance cerebrovascular health, *Hibiscus* may also contribute to the prevention of cognitive decline and dementia, although this requires confirmation in long-term neurocognitive trials. Its excellent safety and tolerability profile make *Hibiscus* an attractive candidate for integrative cardiometabolic and neuroprotective interventions. *Hibiscus sabdariffa* demonstrates antioxidant and anti-inflammatory effects through regulation of oxidative stress pathways and cytokine production. Nonetheless, its therapeutic application is constrained by limited direct neuroclinical data and variability in extract composition [90–93].

#### *Camellia sinensis* (Green Tea)

Polyphenols in *Camellia sinensis*, particularly epigallocatechin-3-gallate (EGCG), have been extensively studied for their metabolic and neurological effects. EGCG enhances glucose uptake via GLUT4 translocation, improves insulin sensitivity, and exerts mild diuretic actions [94]. Epidemiological studies associate regular green tea consumption with reduced risk of T2DM and improved cognitive function in elderly populations [95]. Neuroprotective mechanisms include antioxidant activity, mitochondrial stabilization, and inhibition of amyloid-β aggregation [96]. Although some RCTs report modest effects on glycemic control, cumulative evidence supports a beneficial role of green tea polyphenols in cardiometabolic health and neurodegeneration prevention. The bioactive compounds in *Camellia sinensis*, particularly catechins, exert neuroprotective effects by reducing oxidative stress and modulating inflammatory signaling pathways. However, more robust clinical trials are needed to validate these effects in neurological conditions [94, 95, 97].

#### *Cinnamomum verum* (Cinnamon)

*Cinnamomum verum* and related species contain polyphenols that mimic insulin action, thereby improving insulin sensitivity and lowering blood glucose. Meta-analyses of clinical trials demonstrate modest but significant reductions in fasting glucose and HbA1c, with greater benefits in individuals with poor baseline glycemic control [98, 99]. Beyond metabolic effects, cinnamon polyphenols exhibit strong antioxidant and anti-inflammatory activity, which may contribute to neuroprotective outcomes, though robust evidence from clinical cognitive trials is still lacking. Safety considerations include coumarin content, which is higher in *Cinnamomum cassia* than in *C. verum*. Overall, cinnamon supplementation appears to be a safe, low-cost adjunct to standard diabetic therapy with potential neurological benefits. *Cinnamomum verum* exerts its effects through modulation of insulin signaling and reduction of oxidative stress and inflammatory pathways. However, its clinical efficacy remains variable because of differences in dosage, formulation, and the limited number of large-scale human studies [98, 99].

#### Berberine (from Coptis/Berberis spp.)

Berberine, an isoquinoline alkaloid derived from *Coptis chinensis* and *Berberis* species, exhibits pharmacological properties comparable to metformin. It activates AMPK, enhances glycolysis, and reduces hepatic gluconeogenesis, leading to significant improvements in fasting glucose, HbA1c, and lipid profiles in patients with T2DM [100]. Preclinical studies further reveal anticonvulsant and anti-inflammatory actions through modulation of GABAergic signaling and suppression of oxidative stress in seizure models [101]. Clinical trials support its efficacy at doses of 500 mg three times daily, although gastrointestinal intolerance and drug–drug interactions mediated by P-glycoprotein and cytochrome P450 isoenzymes should be considered [102]. Berberine’s dual metabolic and neuroprotective activities underscore its relevance as a plant-derived prototype for drug development. Berberine demonstrates neuroprotective and metabolic effects primarily through activation of AMPK and modulation of inflammatory and oxidative stress pathways, contributing to improved cellular energy metabolism. Despite promising findings, its clinical use is limited by low bioavailability and the need for further well-controlled clinical trials [100–102] (Table 2).

**Table 2:** Medicinal plants with dual metabolic and neuroprotective effects

Plant	Key bioactive compounds	Metabolic effects	Neurological effects	Main evidence source	Ref
*Nigella sativa*	Thymoquinone	↓ glucose, AMPK activation, antioxidant activity	Anticonvulsant and antioxidant effects	Animal studies support anticonvulsant mechanisms; limited human clinical evidence includes metabolic studies and a pilot epilepsy study	[62–66]
*Moringa oleifera*	Flavonoids, glucosinolates	↓ hyperglycemia, ↑ insulin sensitivity	Anxiolytic and anticonvulsant effects	Mainly animal evidence for neurological effects; limited human clinical evidence for metabolic outcomes	[85–88]
*Curcuma longa*	Curcumin	↓ inflammation, ↑ insulin sensitivity	Antidepressant, anti-amyloid, and neuroprotective effects	Mixed evidence; human clinical studies support effects on depression and selected cognitive outcomes, while many mechanisms remain preclinical	[67–70]
*Salvia officinalis*	Essential oils, polyphenols	↑ insulin sensitivity, α-glucosidase inhibition	Cognitive enhancement and anticonvulsant potential	Human clinical evidence exists for cognitive outcomes in Alzheimer’s disease; anticonvulsant and mechanistic effects are mainly preclinical	[82–84]
*Hibiscus sabdariffa*	Anthocyanins, hibiscus acid	↓ blood pressure, ↓ glucose, improved vascular function	Potential neurovascular protection	Human clinical evidence supports cardiometabolic effects; direct neurological evidence remains limited and mainly indirect	[90–93]
*Camellia sinensis*	EGCG and catechins	↑ glucose uptake, improved insulin sensitivity	Anti-amyloid and neuroprotective effects	Human observational and pilot evidence exists for cognitive associations; anti-amyloid and mitochondrial mechanisms are mainly preclinical	[94–96]
*Cinnamomum verum*	Polyphenols	↓ HbA1c, ↑ insulin sensitivity	Antioxidant and potential neuroprotective effects	Human clinical evidence supports metabolic effects; direct neurological evidence is limited and mainly preclinical or mechanistic	[98, 99]
Berberine	Isoquinoline alkaloid	AMPK activation, ↓ glucose, improved lipid profile	Anticonvulsant and anti-inflammatory effects	Human clinical evidence supports metabolic effects; neurological effects are mainly based on animal epilepsy models	[100–102]

#### Negative and Conflicting Evidence for Medicinal Plants

Although the medicinal plants discussed in this review show promising metabolic and neuroprotective effects, the available evidence is not uniformly positive. For *Moringa oleifera*, a randomized placebo-controlled clinical trial in therapy-naïve patients with type 2 diabetes reported no significant improvement in fasting plasma glucose or HbA1c after short-term supplementation, although the intervention was well tolerated [88]. For Curcuma longa, clinical findings are influenced by differences in curcumin formulation, dose, bioavailability, treatment duration, and patient population; therefore, positive findings in depression or cognition should not be generalized to all neurological disorders [68, 70]. Similarly, although cinnamon supplementation has shown modest metabolic benefits in some studies, the results across trials and meta-analyses remain heterogeneous, with variability related to cinnamon species, preparation, dose, baseline glycemic status, and study duration [98, 99]. Direct human neurological evidence also remains limited for several botanicals, including *Hibiscus sabdariffa*, *Moringa oleifera*, cinnamon, and berberine. Therefore, the neuroprotective potential of these medicinal plants should be interpreted cautiously, particularly when findings are derived mainly from animal models, short-term trials, or indirect cardiometabolic outcomes rather than robust neurological clinical endpoints.

### Probiotics as Dual Modulators of the Gut–Brain–Metabolic Axis

In addition to medicinal plants, probiotics are increasingly recognized as biological modulators with pleiotropic actions that extend beyond gastrointestinal health to include significant metabolic and neurological benefits. The emerging concept of psychobiotics describes live microorganisms that, when administered in adequate amounts, confer mental health benefits by modulating the gut–brain axis [103] (Table 3).

**Table 3:** Probiotics and their neuro-metabolic mechanisms

Mechanism	Effects	Clinical/Neurological Relevance	Main evidence source	Ref
SCFA production	↑ butyrate, propionate	Improves insulin sensitivity and reduces inflammation	Human metabolic studies and preclinical gut–brain axis evidence	[71, 72]
Neurotransmitter production	↑ GABA, serotonin, dopamine	May improve mood, cognition, and neuronal signaling	Mainly preclinical evidence, with limited indirect human support	[74, 75]
HPA axis regulation	↓ cortisol	May reduce stress and depressive symptoms	Human clinical and preclinical evidence	[75, 104]
Anti-inflammatory	↓ TNF-α, IL-6	May reduce systemic inflammation and neuroinflammatory signaling	Mixed evidence; human clinical support is stronger for depression/metabolic outcomes than for direct neurological disease modification	[74, 75]
Gut barrier integrity	↓ permeability	May reduce systemic inflammation and indirectly protect CNS function	Mainly preclinical evidence with supporting human metabolic data	[71]

#### Metabolic Actions

Probiotics exert beneficial metabolic effects by improving glucose tolerance, enhancing insulin sensitivity, lowering serum lipids, and modulating blood pressure. Mechanistically, they alter gut microbiota composition, increase short-chain fatty acid (SCFA) production (notably butyrate and propionate), strengthen intestinal barrier integrity, and reduce systemic inflammation [71]. The RCTs and meta-analyses have demonstrated significant improvements in fasting blood glucose, HbA1c, and insulin resistance indices in patients with T2DM following supplementation with multi-strain probiotics [72]. Probiotics also regulate sodium absorption and fluid balance through effects on intestinal transporters, which may contribute to reductions in blood pressure [73].

#### Neurological and Neuropsychiatric Actions

**Fig. 3:**
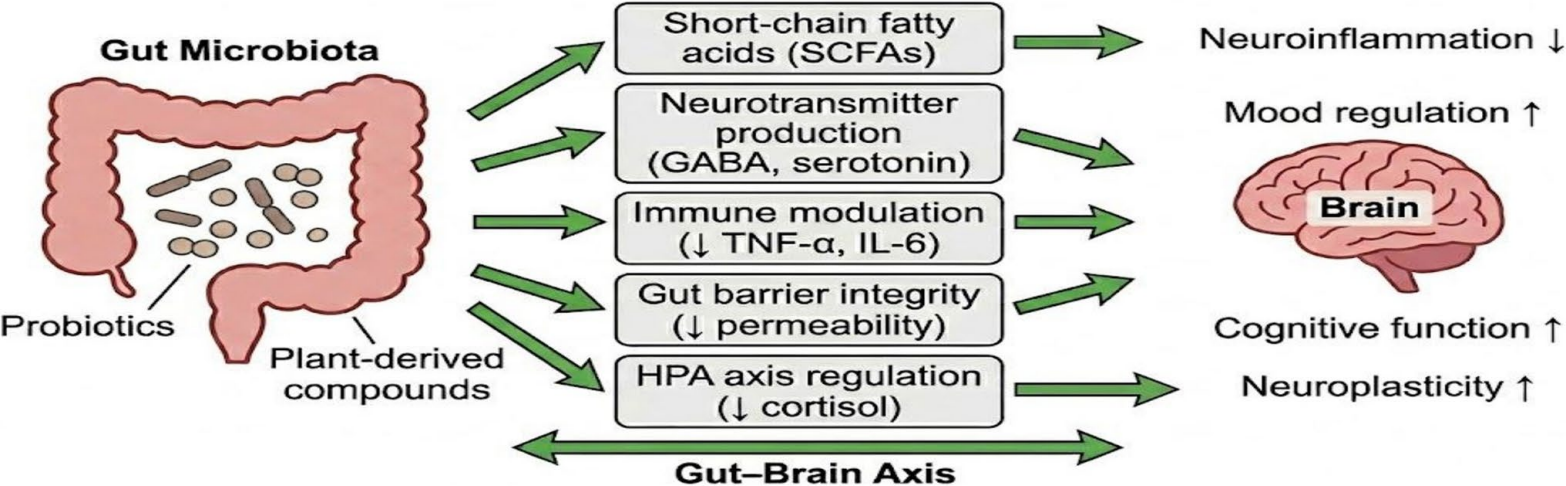
Modulation of the gut–brain axis by probiotics and plant-derived natural products. Probiotics and plant-derived compounds influence brain function through the gut–brain axis via multiple interconnected pathways, including the production of short-chain fatty acids, modulation of neurotransmitters such as GABA and serotonin, regulation of immune responses, maintenance of gut barrier integrity, and attenuation of hypothalamic–pituitary–adrenal (HPA) axis activity. These mechanisms collectively contribute to improved cognitive function, enhanced mood regulation, reduced neuroinflammation, and increased neuroplasticity

On the neurological side, probiotics modulate the gut–brain axis through multiple interconnected mechanisms, including neurotransmitter production, immune regulation, and HPA axis modulation (Fig. 3). They produce neurotransmitters and neuromodulators, including GABA, serotonin, and dopamine, which influence central nervous system signaling. They also regulate the HPA axis, lowering cortisol levels and attenuating stress responses. In addition, probiotics reduce neuroinflammation by suppressing microglial activation and decreasing pro-inflammatory cytokines such as TNF-α and IL-6, while enhancing neuroplasticity partly through increases in BDNF levels [75, 105]. Clinical trials substantiate these mechanistic insights: supplementation with *Lactobacillus plantarum* PS128 in children with ASD improved social responsiveness and reduced hyperactivity [106], while probiotic interventions in patients with MDD led to significant reductions in Hamilton Depression Rating Scale scores and improvements in mood biomarkers [104]. Additional trials and meta-analyses confirm that probiotics exert anxiolytic and antidepressant effects, particularly in populations with elevated baseline symptoms [107, 108]. Preclinical evidence also indicates anticonvulsant activity, with studies showing normalization of hippocampal excitability and restoration of glutamate/GABA balance in epilepsy models [109, 110]. Moreover, animal and human studies in AD suggest that probiotics may reduce amyloid-beta deposition, mitigate oxidative stress, and enhance cognitive performance [111, 112]. Taken together, these findings suggest that probiotics may have potential as psychobiotics and neuro-metabolic adjuncts; however, the clinical evidence is not uniformly positive and appears to be strain-specific, population-specific, and outcome-dependent. For example, a double-blind randomized placebo-controlled trial of *Lactobacillus helveticus* and *Bifidobacterium longum* in participants with depressive symptoms found no significant difference between probiotic and placebo groups in psychological outcomes or blood-based biomarkers [108]. In Alzheimer’s disease and mild cognitive impairment, probiotic studies also remain heterogeneous, with some analyses suggesting benefits mainly in mild cognitive impairment rather than established Alzheimer’s disease, while other findings remain inconclusive because of small sample sizes, different strains, variable treatment durations, and differences in cognitive assessment tools [111, 112, 112–114]. Therefore, probiotics should currently be considered promising adjunctive interventions rather than established disease-modifying treatments for neurological or psychiatric disorders. These mechanisms overlap substantially with those described for plant-derived bioactive compounds, supporting a possible complementary role for probiotics and medicinal plants in modulating oxidative stress, inflammation, and metabolic dysfunction, but this role requires further validation in large, well-designed randomized controlled trials [71–75, 103, 106–112, 114].

#### Translational Promise

The dual modulation of metabolic and neurological pathways positions probiotics as attractive adjunctive therapies for complex comorbid conditions, such as T2DM with depression or metabolic syndrome with cognitive decline. However, heterogeneity in strain selection, dosage, and study design underscores the need for standardized, large-scale RCTs to confirm long-term efficacy and safety.

### Summary of Evidence Strength for Medicinal Plants and Probiotics

To improve interpretation of the translational relevance of the reviewed interventions, the strength of evidence for each medicinal plant and probiotic intervention is summarized in Table 4. Overall, the evidence base is strongest for metabolic and cardiometabolic outcomes, whereas direct neurological or psychiatric clinical evidence remains more limited. Several interventions are supported mainly by animal or mechanistic studies, while only selected compounds or probiotic preparations have been evaluated in human neurological or psychiatric populations. Therefore, the findings should be interpreted according to the source and strength of evidence rather than assuming that all mechanistic effects are clinically established.

**Table 4:** Strength of evidence for medicinal plants and probiotics discussed in this review

Intervention	Main evidence source	Strength for metabolic/cardiometabolic outcomes	Strength for neurological/psychiatric outcomes	Key interpretation and limitation	Ref
*Salvia officinalis*	Human clinical studies and preclinical mechanistic studies	Moderate	Limited to moderate for cognition; mainly preclinical for anticonvulsant effects	Evidence supports potential cognitive and metabolic benefits, but neurological evidence is based on limited clinical studies and requires larger trials	[82–84]
*Nigella sativa*/thymoquinone	Human metabolic studies, animal seizure models, and a small pilot epilepsy study	Moderate	Limited to moderate	Metabolic evidence is supported by human and experimental studies, while epilepsy-related evidence includes animal data and a small pilot clinical trial; larger RCTs are needed	[62, 63, 65, 66]
*Moringa oleifera*	Mainly animal studies with limited human metabolic trials	Limited and partly inconsistent	Low/preclinical	Neurological effects are mainly supported by animal studies. Human metabolic evidence is limited and not uniformly positive, so clinical translation remains uncertain	[85, 86, 88]
*Curcuma longa*/curcumin	Human clinical studies, systematic reviews, and extensive preclinical evidence	Moderate	Moderate for depressive symptoms and selected cognitive outcomes; limited for disease modification	Curcumin has supportive human evidence in depression and selected cognitive outcomes, but poor bioavailability, formulation differences, and heterogeneous trial designs limit generalization	[67–70]
*Hibiscus sabdariffa*	Human cardiometabolic trials and preclinical antioxidant/anti-inflammatory evidence	Moderate	Low/indirect	Clinical evidence supports blood pressure and cardiometabolic effects, but direct neurological clinical evidence is lacking; neuroprotective relevance is mainly indirect through vascular and metabolic pathways	[90, 90–93]
*Camellia sinensis*/EGCG	Human metabolic and observational/pilot cognitive studies plus preclinical neuroprotective evidence	Limited to moderate	Limited to moderate	Green tea catechins show antioxidant and anti-amyloid mechanisms, with some human cognitive and metabolic data; however, direct neurological clinical evidence remains limited	[94, 94, 97]
*Cinnamomum verum*/cinnamon	Human metabolic trials and meta-analyses; limited mechanistic neurological evidence	Moderate but heterogeneous	Low/indirect	Evidence is stronger for glycemic outcomes than for neurological effects. Clinical results vary by cinnamon species, dose, preparation, and study duration	[98, 99]
Berberine	Human metabolic studies and animal neurological models	Moderate	Low/preclinical	Human evidence supports metabolic effects, while neurological evidence is mainly derived from animal epilepsy and inflammation models. Low bioavailability and interaction potential remain important limitations	[100–102]
Multi-strain probiotics for metabolic regulation	Human metabolic RCTs and mechanistic gut–brain axis studies	Moderate	Low/indirect	Clinical evidence supports some metabolic effects, but direct neurological benefit is not established for most metabolic probiotic preparations	[71–73]
*Lactobacillus plantarum* PS128	Human randomized controlled trial in children with ASD	Not primarily evaluated	Limited to moderate	Evidence suggests possible improvement in selected ASD-related behavioral outcomes, but support is based on limited clinical data and requires replication in larger multicenter trials	[106]
Probiotic/prebiotic interventions in MDD	Human clinical trials and systematic reviews	Not primarily evaluated	Moderate but heterogeneous	Some studies report improvement in depressive symptoms, while others show neutral findings. Effects appear strain-specific and population-dependent	[75, 104, 107, 108]
*Lactobacillus helveticus* and *Bifidobacterium longum*	Human randomized placebo-controlled trial in depressive symptoms	Not primarily evaluated	Limited/inconsistent	One randomized trial did not show significant benefit over placebo, indicating that probiotic effects on mood are not uniform across strains or populations	[108]
Probiotics in epilepsy	Animal models and limited pilot human evidence	Not primarily evaluated	Low to limited	Evidence includes preclinical support and a small pilot study in drug-resistant epilepsy, but robust randomized clinical evidence is lacking	[109, 110]
Probiotics in Alzheimer’s disease and cognitive impairment	Animal studies and small human clinical studies	Limited	Limited and heterogeneous	Findings suggest possible cognitive and metabolic benefits in selected studies, but evidence remains limited by small sample sizes, strain heterogeneity, and variable cognitive outcomes	[111, 112]

## Comparative and Translational Perspectives

Neurological and psychiatric disorders are increasingly recognized as multifactorial conditions driven by interconnected pathways, including oxidative stress, neuroinflammation, metabolic dysregulation, and impaired neuroplasticity. Conventional pharmacological approaches, such as SGLT2 inhibitors, have demonstrated pleiotropic effects that extend beyond their primary metabolic functions, offering neuroprotective benefits by modulating shared mechanisms. In parallel, medicinal plants and probiotics have emerged as promising complementary strategies, exhibiting multi-target actions that align closely with these pharmacological pathways.

The comparative analysis presented in Table 5 highlights the substantial overlap in mechanisms between SGLT2 inhibitors, plant-derived bioactive compounds, and probiotics. Despite differences in origin and primary therapeutic use, these interventions converge on key biological processes. For instance, all three approaches effectively attenuate oxidative stress by enhancing endogenous antioxidant defenses and reducing reactive oxygen species. Similarly, suppression of neuroinflammation is a shared feature, mediated by inhibition of pro-inflammatory signaling pathways such as NF-κB and by reductions in cytokines, including TNF-α and IL-6.

**Table 5:** Comparative mechanisms of SGLT2 inhibitors, medicinal plants, and probiotics

Mechanism	SGLT2 Inhibitors	Medicinal Plants	Probiotics
Oxidative stress	↓ ROS, ↑ antioxidants	Polyphenols, flavonoids	SCFA-mediated antioxidant effects
Neuroinflammation	↓ NF-κB, cytokines	Anti-inflammatory phytochemicals	↓ cytokines, immune modulation
Metabolism	↓ glucose, ↑ ketones	AMPK activation	↑ insulin sensitivity
Neurotransmission	↑ BDNF, membrane stability	GABA modulation, neuroprotection	↑ neurotransmitter production
Gut–brain axis	Indirect modulation	Partial modulation	Direct regulation

Metabolic regulation represents another critical point of convergence. While SGLT2 inhibitors directly reduce plasma glucose levels and promote ketogenesis, medicinal plants often exert similar effects by activating AMP-activated protein kinase (AMPK) and modulating insulin signaling. Probiotics complement these actions by improving insulin sensitivity and producing short-chain fatty acids that influence host metabolism. These overlapping metabolic effects are particularly relevant given the strong association between metabolic dysfunction and neurological disorders.

In addition, all three modalities influence neuronal function and synaptic plasticity. SGLT2 inhibitors enhance brain-derived neurotrophic factor (BDNF) expression and stabilize neuronal membranes, while plant-derived compounds modulate neurotransmitter systems and protect against excitotoxicity. Probiotics further contribute through the gut–brain axis by producing neuroactive compounds such as GABA and serotonin and regulating hypothalamic–pituitary–adrenal (HPA) axis activity.

Importantly, probiotics provide a unique advantage through direct modulation of the gut–brain axis, a pathway increasingly implicated in neurological and psychiatric disorders. While SGLT2 inhibitors and medicinal plants may exert indirect effects on this axis, probiotics actively reshape gut microbiota composition, strengthen intestinal barrier integrity, and influence neuroimmune signaling.

Collectively, these findings support a translational framework in which plant-derived natural products, probiotics, and SGLT2 inhibitors may act on overlapping biological pathways. Nevertheless, this convergence of mechanisms does not by itself demonstrate clinical synergy. To date, the evidence base mainly consists of separate studies evaluating individual medicinal plants, individual probiotic preparations, or SGLT2 inhibitors, whereas direct clinical trials evaluating combined use are limited or absent. Consequently, any synergistic effect among these interventions should be described as theoretical, mechanistically plausible, and investigational. Future studies should directly compare monotherapy with combined regimens, include clinically meaningful neurological and psychiatric endpoints, and monitor adverse events, pharmacokinetic interactions, glycemic effects, microbiota changes, and long-term safety before such combinations can be recommended in clinical practice.

## Limitations and Future Perspectives

Despite encouraging mechanistic and preclinical findings, several important limitations restrict the immediate use of plant-derived compounds and probiotics in routine clinical practice for neurological and psychiatric disorders. First, much of the available evidence is derived from in vitro studies, animal models, observational studies, or small pilot clinical trials, whereas large, well-designed randomized controlled trials in clearly defined neurological or psychiatric populations remain limited. Therefore, antioxidant, anti-inflammatory, mitochondrial, anticonvulsant, antidepressant, or cognitive effects observed in experimental models should not be directly interpreted as established clinical efficacy in humans.

A second major limitation is the heterogeneity and lack of standardization of plant-derived interventions. The concentration of bioactive compounds may vary according to plant species, geographical origin, cultivation conditions, extraction method, processing, storage, and formulation. This variability makes it difficult to compare studies, define optimal therapeutic doses, reproduce clinical effects, or develop consistent treatment protocols. In addition, several phytochemicals, such as curcumin and berberine, have limited oral bioavailability, rapid metabolism, or uncertain blood–brain barrier penetration, which may reduce their clinical effectiveness despite strong mechanistic activity in preclinical studies.

Safety and interaction issues also limit clinical translation. Although many medicinal plants are perceived as safe, they may still cause adverse effects, gastrointestinal intolerance, hepatotoxicity, allergic reactions, or clinically relevant herb–drug interactions, particularly in patients receiving antiepileptic drugs, antidepressants, anticoagulants, antidiabetic agents, antihypertensive drugs, or other long-term medications. For example, differences in cinnamon species may influence coumarin exposure, while berberine may interact with drug transporters and metabolizing enzymes. Product adulteration, contamination, inaccurate labeling, and inconsistent quality control are additional concerns, especially for non-standardized commercial supplements.

Probiotics also face important clinical limitations. Their effects are strain-specific and cannot be generalized across all probiotic products. Clinical outcomes may depend on the administered strain, dose, treatment duration, viability of the preparation, storage conditions, baseline gut microbiota composition, diet, age, disease stage, antibiotic exposure, and concomitant medications. In addition, although probiotics are generally well tolerated, caution is required in immunocompromised patients, critically ill patients, premature infants, and individuals with central venous catheters or severe intestinal barrier dysfunction because of the potential risk of infection or other adverse events. Long-term safety data in neurological and psychiatric populations remain insufficient.

Another limitation is that most studies evaluate short-term biomarker changes, metabolic outcomes, or symptom scores rather than long-term neurological endpoints such as disease progression, seizure frequency reduction over extended periods, cognitive decline, functional independence, relapse prevention, or quality of life. Furthermore, proposed synergistic effects between medicinal plants, probiotics, and pharmacological agents remain largely theoretical or preclinical, with insufficient clinical evidence to recommend routine combined use. These interventions should therefore be regarded as potential adjunctive strategies rather than replacements for established evidence-based treatments.

Future research should prioritize standardized formulations, validated dosing regimens, pharmacokinetic and pharmacodynamic studies, assessment of BBB penetration, long-term safety monitoring, and adequately powered randomized controlled trials using clinically meaningful neurological and psychiatric endpoints. Regulatory frameworks are also needed to ensure product quality, purity, labeling accuracy, and post-marketing safety surveillance. Interdisciplinary collaboration among neurologists, psychiatrists, endocrinologists, pharmacologists, microbiome researchers, and clinical trial specialists will be essential to translate the promise of plant-derived compounds and probiotics into safe, reproducible, and evidence-based clinical applications.

## Conclusion

Plant-derived natural products represent a mechanistically diverse group of bioactive agents with potential relevance to neurological and psychiatric disorders. Through modulation of oxidative stress, inflammation, mitochondrial function, metabolic pathways, synaptic plasticity, and gut–brain axis signaling, these compounds may influence several disease-relevant mechanisms involved in Alzheimer’s disease, epilepsy, autism spectrum disorder, and major depressive disorder.

Medicinal plants such as *Nigella sativa*, *Moringa oleifera*, Curcuma longa, *Salvia officinalis*, *Hibiscus sabdariffa*, *Camellia sinensis*, cinnamon, and berberine demonstrate promising neuroprotective or neuro-metabolic effects in preclinical and selected clinical studies. However, the strength of evidence varies considerably across interventions. For several compounds, direct neurological evidence remains mainly preclinical or indirect, while stronger human evidence often relates to metabolic or cardiometabolic outcomes rather than confirmed neurological disease modification.

Probiotics and psychobiotic interventions further support the importance of the gut–brain axis as a therapeutic target. Their effects on neurotransmitter production, immune regulation, intestinal barrier integrity, short-chain fatty acid production, and hypothalamic–pituitary–adrenal axis modulation provide a biologically plausible link between microbiota regulation and neurological or psychiatric outcomes. Nevertheless, probiotic effects are strain-specific, and current clinical evidence remains heterogeneous across populations, strains, doses, and outcomes.

SGLT2 inhibitors provide a useful comparative pharmacological model because they share several metabolic, antioxidant, anti-inflammatory, vascular, and potential neuroprotective mechanisms with plant-derived compounds and probiotics. However, they are not plant-derived compounds, and their inclusion in this review should be interpreted as a mechanistic reference point rather than as a botanical intervention.

Overall, the current evidence supports a promising but still developing framework for plant-derived natural products, probiotics, and metabolically active pharmacological models in neurological and psychiatric disorders. Proposed synergistic effects between medicinal plants, probiotics, and SGLT2 inhibitors remain mechanistically plausible but are not yet supported by sufficient direct clinical evidence. Therefore, these interventions should currently be considered potential adjunctive or investigational strategies rather than replacements for established evidence-based treatments. Future research should focus on standardized formulations, improved bioavailability, rigorous safety assessment, and adequately powered randomized controlled trials with clinically meaningful neurological and psychiatric endpoints.

## Data Availability

No datasets were generated or analysed during the current study.

## Abbreviations

MDD: Major Depressive Disorder
ASD: Autism Spectrum Disorder
AD: Alzheimer's Disease
SGLT2: Sodium-glucose cotransporter-2
Aβ: Amyloid-beta
GSH: Glutathione
SOD: Superoxide dismutase
BBB: Blood-brain barrier
ROS: Reactive oxygen species
NF-κB: Nuclear factor κ-light chain enhancer of activated B cells
T2DM: Type 2 diabetes mellitus
UGE: Urine glucose excretion
TNF-α: Tumor Necrosis Factor-alpha
IL-6: Interleukin-6
PET: Positron Emission Tomography
NAFLD: Non-alcoholic fatty liver disease
RAAS: Renin-angiotensin-aldosterone system
eGFR: Estimated glomerular filtration rate
NFTs: Neurofibrillary tangles
APP: Amyloid precursor protein
CSF: Cerebrospinal fluid
IL-1β: Interleukin-1 beta
PTZ: Pentylenetetrazol
BDNF: Brain-derived neurotrophic factor
AEDs: Antiepileptic drugs
RCTs: Randomized controlled trials
NaV: Voltage-gated sodium
KV: Voltage-gated potassium
CaV: Voltage-gated calcium
NMDA: N-methyl-D-aspartate
AMPA: Alpha-amino-3-hydroxy-5-methyl-4-isoxazolepropionic acid
